# Factor VIII Activity in Relation to the Type of Thrombosis and Patient’s Risk Factors for Thrombosis, Age, and Comorbidity

**DOI:** 10.5152/eurasianjmed.2023.22072

**Published:** 2023-02-01

**Authors:** Dusica Basaric, Marko Saracevic, Vesna Bosnic, Anka Vlatkovic, Branko Tomic, Mirjana Kovac

**Affiliations:** 1 Blood Transfusion Institute of Serbia, Hemostasis Department, Serbia, Belgrade; 2 Institute of Molecular Genetics and Genetic Engineering, University of Belgrade, Serbia, Belgrade; 3 Faculty of Medicine, University of Belgrade, Serbia, Belgrade

**Keywords:** Factor VIII activity, thrombosis type, age, comorbid diseases

## Abstract

**Objective:**

Elevated factor VIII has been shown to be an independent risk factor for deep venous thrombosis and pulmonary embolism. It has been suggested that increased factor VIII levels by itself is insufficient to cause thrombosis; however, increased factor VIII with other risk factors could increase the risk of thrombosis. The aim of the study was to evaluate the factor VIII level with regard to the type of thrombosis and patient’s risk factors such as age or comorbidity.

**Materials and Methods:**

In total, 441 patients who were referred for thrombophilia testing from the period of January 2010 to December 2020 were included in the study. The patients who developed the first thrombosis before the age of 50 were eligible for the study. The patients’ data that were used in statistical analyses were collected from our thrombophilia register.

**Results:**

The number of the subjects with increased factor VIII over 1.5 IU/mL is equal regardless of the thrombosis type. Factor VIII activity already begins to increase over 40 years old and reaches the mean values of 1.45 IU/mL close to the cut-off (1.5 IU/mL), showing a statistically significant difference compared to those under 40, *P*  = .001. Comorbidities other than thyroid disease or malignancy had no influence on the increase of factor VIII. In the mentioned conditions, the average factor VIII of 1.82 (0.79) and 1.65 (0.43) was obtained, respectively.

**Conclusion:**

Factor VIII activity is significantly affected by age. Thrombosis type and comorbid diseases other than thyroid disease and malignancy had no effect on factor VIII.

**Main Points**

- The increase in factor VIII (FVIII) activity is significantly affected by age.
- Factor VIII activity has already begun to increase over 40 years old.
- This age-related hemostatic change may contribute to the development of venous thromboembolism.
- With the exception of malignancy or thyroiditis, comorbidities do not affect the increase in FVIII.
- Determination of FVIII could be helpful in the evaluation of the thrombosis risk.

## Introduction

Aging is associated with a number of different hemostatic changes that cause an increase in procoagulant status, which increases the risk of venous thromboembolism (VTE).^1-3^ Additionally, the presence of thrombosis risk factors or contributing triggers for VTE varies widely with regard to different ages and also depends upon the presence of comorbidities.^2^ According to the literature data, the incidence of VTE increases exponentially with the age, with a pointed increase of 1.9-fold per decade.^4^

It is well known that in healthy humans in parallel with the physiological processes of aging, the plasma concentrations of many coagulation factors increase.^1^ Having in mind the association with the risk of thrombosis, special importance is given to factor VIII (FVIII). Elevated FVIII has been shown to be an independent risk factor for deep venous thrombosis (DVT) and/or pulmonary embolism (PE).^5-10^ It has been suggested that increased FVIII levels by itself is insufficient to cause thrombosis; however, increased FVIII with other risk factors could increase the risk of thrombosis.^11,12^ The mechanism by which an increase in FVIII levels leads to increased thrombogenicity has not been fully understood.^8-12^ With regard to the previously conducted studies, FVIII level over 1.5 IU/mL is associated with an approximately 5-fold increased risk of venous thrombosis. Considering its progressive increase related to age, FVIII reaches a mean level of over 2.0 IU/mL in the seventh decade of life.^13-15^ Such an increase is often followed by a shortening of the activated partial thromboplastin time (APTT) that is also a well-established thrombosis risk factor.^16-19^ High factor VIII has also been observed in patients with different types of malignancy.^20-22^ With regard to genetic influence, the best-known genes affecting the determinants of FVIII levels are the ABO blood group and vWF levels.^23^ While, in a very recently published study, Simoni et al^7^ showed that the first thrombophilic defect in the *F8 *gene, designated “FVIII Padua,” was associated with significantly elevated FVIII levels (over 2.5 IU/mL). Considering the important role of FVIII in the hemostatic process, the examination of FVIII activity was performed in all our patients who were referred for thrombophilia testing due to the development of the first thrombosis before the age of 50. With regard to the type of thrombosis, the risk factors that appear as triggers in the development of thrombosis may be different depending on the type of thrombosis. In the case of isolated superficial venous thrombosis (SVT), a varicose vein and malignancy are the most common; while in the case of DVT surgery, trauma, pregnancy, and hormonal use are the most common.^24^ On the other hand, SVT is characterized by the combination of thrombosis and inflammation in a superficial vein and in up to 60%-80% of cases, involves the great saphenous vein.^25^

The aim of this study was to evaluate the FVIII level changes with regard to the type of thrombosis and the patient’s risk factors such as age or comorbidity among those patients who developed thrombosis.

## Materials and Methods

This retrospective study included 441 patients; 221 females and 220 males (average 45 years), who were referred for thrombophilia testing, between January 2010 and December 2020. With regard to the defined indication for the thrombophilia testing, the patients who developed their first thrombosis before the age of 50, confirmed with color Doppler or computer tomography, were eligible for the study. Demographic data related to gender, age, comorbidities, thrombosis characteristics, family history, and laboratory results including FVIII activity, APTT, and fibrinogen level were collected from our laboratory information system and thrombophilia register. The information about comorbidities was obtained from the attached patient documentation on the basis of the obtained anamnestic data at the time of sampling.

For each patient, 2 samples of whole blood were collected into buffered sodium citrate tubes, containing 1/10 volume sodium citrate stock solution at 0.129 mmol/L (Vacutest, Kima, Cell BioSciences, Italy). The samples were prepared by a standard method in order to obtain the platelet-poor plasma for coagulation assays included in the thrombophilia testing that was performed on the Siemens BCS XP Coagulation System. The level of FVIII above 1.5 IU/mL was considered as increased.^11^ With the aim to minimize the influence of acute thrombosis on FVIII level as an acute-phase protein, the determination of FVIII was done in 6-8 weeks period after the acute thrombosis event. In those patients with FVIII activity over 1.5 IU/mL, analyses were repeated 1 month later.^22^

Institutional approval for the study was granted by the Ethics Committee of Blood Transfusion Institute of Serbia, Belgrade (number 1390/2) in accordance with the internationally accepted ethical standards and each patient signed the informed consent form.

### Statistical Analyses

The Statistical Package for Social Sciences 20.0 for Windows (IBM SPSS Corp.; Armonk, NY, USA) was used for statistical analysis. Mann–Whitney *U*-test and Pearson chi-square test were used for characteristics evaluation of the study population and the relationship between hemostasis parameters and the type of thrombosis. For correlation analysis, the Spearman tests were used. The probability of *P*  < .05 was considered statistically significant, except for the Spearman test of correlations, where *P*  < .01 was considered statistically significant.

## Results

Three-quarters of the subjects had DVT. In 21% of all study participants, the recurrence rate of the thrombotic event was recorded. Almost one-quarter of the study participants had comorbidity or a transient risk factor during the first thrombotic event, while thrombophilia testing revealed thrombophilia presence in one-quarter. In 14 (3%) of the study participants who had recurrent thrombosis, the recurrent event was observed in the period of malignant disease presence. The average level of FVIII obtained in the study group was 1.48 (0.66), while in 207 (47%) of them, the level of FVIII was above 1.5 IU/mL (Table 1).

There was no difference between the 2 investigated groups in relation to the APTT and FVIII. Shortening of APTT below 26 seconds was observed in the same number of patients regardless of the thrombosis type (16%). Similarly, half of the patients in both groups had elevated levels of FVIII. With regard to the fibrinogen level over 4 g/L, a statistically significant difference was observed, considering that a higher number of those with DVT had elevated fibrinogen level, *P*  < .001 (Table 2).

The influence of comorbidity and age on FVIII, APTT, and fibrinogen levels has been analyzed. A significant influence of age on FVIII and fibrinogen levels was observed, *P*  < .001, (Table 3).

**Figure 1.**
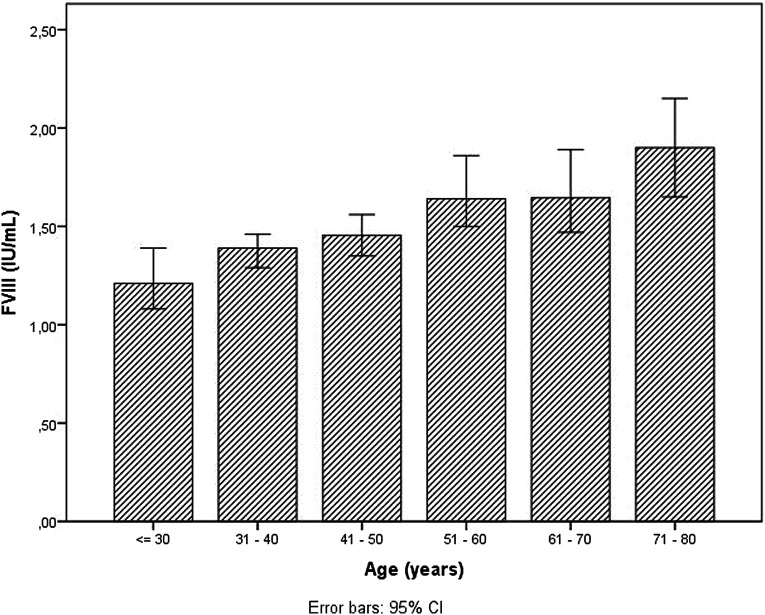
Increase in FVIII levels with regard to the age.

A mean FVIII activity gradually increased with each decade of the age. Factor VIII activity already began to increase in the group over 40 years old reaching the mean value of 1.45 IU/mL, close to the cut-off (1.5 IU/mL), and showed significant difference compared to those under 40, *P*  = .001, (Table 4 and Figure 1).

## Discussion

Our study results showed that the type of thrombosis had no effect on changes in FVIII activity. The average activity of FVIII, as well as the number of subjects with increased levels of FVIII over 1.5 IU/mL, is equal in both groups of subjects regardless of the type of thrombosis (DVT or superficial). In contrast, the increase in FVIII activity is significantly affected by age, while comorbid diseases do not have a significant influence on the level of FVIII activity, except in the case of DVT patients with thyroid or malignant diseases. In the patients with mentioned diseases, FVIII levels of 1.82 (0.79) and 1.65 (0.43) were observed.

Stratification of FVIII levels that was performed in relation to the decade of life showed that in the period after the fifth decade, a significant increase of FVIII occurred. Such an unchanged level of FVIII was maintained until the seventh decade, followed by a new significant increase. Our results are similar to those obtained in the different study populations (industrial or general population) that showed a progressive increase of FVIII with age, reaching a mean of over 2.0 IU/mL in the seventh decade of life.^13-15^ Data from previously published studies showed that the risk of VTE increases continuously and even small changes in FVIII have a major influence.^20,21,26,27^ The risk for a first or recurrent VTE increases by 10% and 24%, respectively, for each 10 IU/dL increment of FVIII activity.^4^

Our study results showed a significant rise in FVIII activity in patients over 40 years old with the mean value of 1.45 IU/mL (close to the cut-off of 1.5 IU/mL). With regard to the hemostatic changes with the age, our analysis of fibrinogen levels also showed a significant increase in fibrinogen that correlates with FVIII activity and age. A higher level of fibrinogen was observed among patients who developed DVT in comparison to those who had superficial thrombosis. The increase in fibrinogen levels seen during aging may explain the increased risk for cardiovascular diseases in elderly people. This has been observed not only in humans but also in experimental animal models.^28^ Tracy et al^29^ showed the rise in fibrinogen at different ages and reported continuous increase in fibrinogen level over the age range 18-85 years. Some studies confirmed that elevated levels of fibrinogen have been correlated with increased risk of cardiovascular events;^30-34^ however, another studies have suggested that the predictive value of fibrinogen may decrease with aging.^35-37^

Bearing in mind the important role they play in the hemostasis system, where fibrinogen contributes to thrombosis, acting as a substrate for thrombus formation, while FVIII plays an important role in the amplification of blood coagulation,^8^ it is important to know the level of their changes depending on age or the presence of comorbid diseases.

However, since fibrinogen and FVIII are acute-phase reactants, it is still debated whether elevated levels of fibrinogen and FVIII only or additionally represent a marker of an enhanced inflammatory state.^32^ Having in mind this fact, in order to minimize the influence of acute thrombosis on the parameters of hemostasis, especially on the level of FVIII as an acute-phase protein, laboratory tests were performed 6-8 weeks after acute thrombosis in all our patients.

Among the limitations that should be discussed is the fact that our study is retrospective, where the long time between the occurrences of the first and recurrent thrombotic events could have an implication that resulted in recall bias. Namely, all included patients developed their first thrombosis before the age of 50; however, some of them were tested after the development of recurrent thrombosis that mostly occurred significantly later in their older age.

However, the clinical significance of this study could be especially important during the decision-making of anticoagulant therapy cessation. Namely, thrombophilia testing can explain the background of a thrombotic event by revealing hemostatic disorders, such as presence of prothrombotic mutations or natural inhibitor deficiency, in less than 50% of the patients, who developed VTE before the age of 50.^38^ While in the remaining patients, the background of the thrombotic event remains unclear. Considering the important role of elevated FVIII activity, as a risk for the first or recurrent thrombosis, the investigation of FVIII among these patients could be helpful in the evaluation of the thrombosis risk presence.

In conclusion, FVIII activity is significantly affected by age. An increase in FVIII was observed in the patients over 40 years old and reached the mean values close to the cut-off (1.5 IU/mL), showing a statistically significant difference compared to those under 40. Thrombosis type and comorbid diseases other than thyroid disease and malignancy had no effect on FVIII. Determination of FVIII could be helpful in the evaluation of the thrombosis risk that is crucial during the decision-making of anticoagulant therapy cessation.

## Figures and Tables

**Table 1.** Characteristics of the Study Population

Number of study participants	441
Age (years), median (IQR)	45 (21)
Male/female	220/221
Deep^*^/superficial	341/100
Recurrent thrombosis, n (%)	95^#^ (21.5)
Comorbidities	91 (20.6)
Cardiovascular disease	15 (3.4)
Diabetes mellitus	12 (2.7)
Thyroid disease^**^	13 (2.9)
Lung disease	4 (0.9)
Kidney disease	3 (0.7)
Liver disease	5 (1.2)
Malignant disease^**^	15 (3.4)
Hyperlipoproteinemia	5 (1.2)
Other	19 (4.3)
Varicose veins	14 (3)
Family history	95 (21.5)
Thrombophilia	111 (25.2)
Transient risk factor for VTE	109 (25)
Surgery	38 (8.6)
Trauma/fracture	32 (7.2)
Pregnancy/postpartum	21 (4.7)
Long sitting position	18 (4)
Oral contraceptives	8 (1.8)
Laboratory results	
FVIII activity, average (IQR)	1.48 (0.66)
APTT	29.5 (5.6)
Fibrinogen	3.2 (1.02)
Patients with	
FVIII > 1.5 IU/mL, n %	207 (47)
APTT < 26 s	70 (15.9)
Fibrinogen > 4.0 g/L	83 (18.8)

^*^62 patients had PE as a complication of DVT, while in 6 patients, the first thrombotic event was isolated PE; ^**^Elevated FVIII levels of 1.82 (0.79) and 1.65 (0.43) were obtained in DVT patients with thyroid disease and malignant disease.

DVT, deep venous thrombosis; PE, pulmonary embolism; APTT, activated partial thromboplastin time; FVIII, factor VIII; IQR, interquartile range.

**Table 2.** Factor VIII Activity, APTT, and Fibrinogen Level in Relation to Thrombosis Type

	Deep Venous Thrombosis	Superficial Venous Thrombosis	*P*
FVIII, average (IQR)	1.5 (0.64)	1.39 (0.66)	.095
Number of patients with FVIII			
≤1.50 IU/mL	171	55	.421
≥1.51 IU/mL	164	43	
APTT, average (IQR)	29.45 (5.6)	29.5 (5.2)	.954
Number of patients with APTT			
< 26.0 seconds	54	16	.665
> 26.0 seconds	263	83	
Fibrinogen, average (IQR)	3.3 (1.1)	3.2 (0.95)	.384
Number of patients with fibrinogen			
2.0-4.0 g/L	163	186	<.001
> 4.0 g/L	55	28	

FVIII, factor VIII; IQR, interquartile range; APTT, activated partial thromboplastin time.

**Table 3.** The Influence of Age and Comorbidities on Factor VIII, APTT, and Fibrinogen Levels

	*P*_(F VIII)_	*P*_(APTT)_	*P*_(Fibrinogen)_
Comorbidities	.774	.879	.059
Age	**<**.001	.813	<.001

FVIII, factor VIII; APTT, activated partial thromboplastin time.

**Table 4.** Factor VIII Activity in Relation to Age Expressed in Decades

Age	N	FVIII (IQR)	*P *				
			≤30	31-40	41-50	51-60	61-70
≤30	55	1.21 (0.54)					
31-40	101	1.39 (0.59)	.036				
41-50	106	1.45(0.57)	.001	.098			
51-60	69	1.64(0.60)	<.001	<.001	.015		
61-70	60	1.65(0.76)	<.001	<.001	.013	.906	
71-80	16	1.9 (0.5)	<.001	<.001	<.001	.039	.075

FVIII, factor VIII; IQR, interquartile range.

## References

[1] Mari D Coppola R Provenzano R . Hemostasis factors and aging. Exp Gerontol. 2008;43(2):66 73. 10.1016/j.exger.2007.06.014) 17869046

[2] Silverstein RL Bauer KA Cushman M Esmon CT Ershler WB Tracy RP . Venous thrombosis in the elderly: more questions than answers. Blood. 2007;110(9):3097 3101. 10.1182/blood-2007-06-096545) 17684155

[3] Wilkerson WR Sane DC . Aging and thrombosis. Semin Thromb Hemost. 2002;28(6):555 568. 10.1055/s-2002-36700) 12536349

[4] Tsai AW Cushman M Rosamond WD Heckbert SR Polak JF Folsom AR . Cardiovascular risk factors and venous thromboembolism incidence. The Longitudinal Investigation of thromboembolism Etiology. Arch Intern Med. 2002;162(10):1182 1189. 10.1001/archinte.162.10.1182) 12020191

[5] Kyrle PA Minar E Hirschl M et al. High plasma levels of factor VIII and the risk of recurrent venous thromboembolism. N Engl J Med. 2000;343(7):457 462. 10.1056/NEJM200008173430702) 10950667

[6] Franchini M Hemostasis and aging. Crit Rev Oncol Hematol. 2006;60(2):144 151. 10.1016/j.critrevonc.2006.06.004) 16860994

[7] Simioni P Cagnin S Sartorello F et al. Partial *F8* gene duplication (factor VIII Padua) associated with high factor VIII levels and familial thrombophilia. Blood. 2021;137(17):2383 2393. 10.1182/blood.2020008168) 33275657

[8] Jenkins PV Rawley O Smith OP O'Donnell JS . Elevated factor VIII levels and risk of venous thrombosis. Br J Haematol. 2012;157(6):653 663. 10.1111/j.1365-2141.2012.09134.x) 22530883

[9] Vlot AJ Koppelman SJ van den Berg MH Bouma BN Sixma JJ . The affinity and stoichiometry of binding of human factor VIII to von ­Willebrand factor. Blood. 1995;85(11):3150 3157. 10.1182/blood.V85.11.3150.bloodjournal85113150) 7756647

[10] Lollar P Hill-Eubanks DC Parker CG . Association of the factor VIII light chain with von ­Willebrand factor. J Biol Chem. 1988;263(21):10451 10455. 10.1016/S0021-9258(19)81537-5) 3134349

[11] Koster T Blann AD Briët E Vandenbroucke JP Rosendaal FR . Role of clotting factor VIII in effect of von Willebrand factor on occurrence of deep-vein thrombosis. Lancet. 1995;345(8943):152 155. 10.1016/s0140-6736(95)90166-3) 7823669

[12] Kraaijenhagen RA in’t Anker PS Koopman MM et al. High plasma concentration of factor VIII:c is a major risk factor for venous thromboembolism. Thromb Haemost. 2000;83(1):5 9. 10669145

[13] Balleisen L Bailey J Epping PH Schulte H van de Loo J . Epidemiological study on factor VII, factor VIII and fibrinogen in an industrial population: I. Baseline data on the relation to age, gender, body-weight, smoking, alcohol, pill-using, and menopause. Thromb Haemost. 1985;54(2):475 479. 10.1055/s-0038-1657877) 3936217

[14] Lowe GD Rumley A Woodward M et al. Epidemiology of coagulation factors, inhibitors and activation markers: the Third Glasgow Monica Survey. I. Illustrative reference ranges by age, sex and hormone use. Br J Haematol. 1997;97(4):775 784. 10.1046/j.1365-2141.1997.1222936.x) 9217176

[15] Conlan MG Folsom AR Finch A et al. Associations of factor VIII and von Willebrand factor with age, race, sex, and risk factors for atherosclerosis. The atherosclerosis risk in communities (ARIC) Study. Thromb Haemost. 1993;70(3):380 385. 10.1055/s-0038-1649589) 8259533

[16] Lippi G Meschi T Borghi L . Variation of activated partial thromboplastin time according to age and sex in a large population study: analytical and clinical implications. Blood Coagul Fibrinolysis. 2012;23(2):177 178. 10.1097/MBC.0b013e32834ee0fb) 22270798

[17] Mina A Favaloro EJ Mohammed S Koutts J . A laboratory evaluation into the short activated partial thromboplastin time. Blood Coagul Fibrinolysis. 2010;21(2):152 157. 10.1097/MBC.0b013e3283365770) 20051842

[18] Lippi G Salvagno GL Ippolito L Franchini M Favaloro EJ . Shortened activated partial ­thromboplastin time: causes and management. Blood Coagul Fibrinolysis. 2010;21(5):459 463. 10.1097/mbc.0b013e328338dbe8) 20614573

[19] Korte W Clarke S Lefkowitz JB . Short activated partial thromboplastin times are related to increased thrombin generation and an increased risk for thromboembolism. Am J Clin Pathol. 2000;113(1):123 127. 10.1309/g98j-ana9-rmnc-xlyu) 10631865

[20] Yigit E Gönüllü G Yücel I Turgut M Erdem D Cakar B . Relation between hemostatic parameters and prognostic/predictive factors in breast cancer. Eur J Intern Med. 2008;19(8):602 607. 10.1016/j.ejim.2007.06.036) 19046726

[21] Auwerda JJ Sonneveld P de Maat MP Leebeek FW . Prothrombotic coagulation abnormalities in patients with newly diagnosed multiple myeloma. Haematologica. 2007;92(2):279 280. 10.3324/haematol.10454) 17296591

[22] Kovac M Kovac Z Tomasevic Z et al. Factor V Leiden mutation and high FVIII are associated with an increased risk of VTE in women with breast cancer during adjuvant tamoxifen - Results from a prospective, single center, case control study. Eur J Intern Med. 2015;26(1):63 67. 10.1016/j.ejim.2014.12.015) 25592075

[23] O'Donnell J Laffan MA . The relationship between ABO histo-blood group, factor VIII and vonWillebrand factor. Transfus Med. 2001;11(4):343 351. 10.1046/j.1365-3148.2001.00315.x) 11532189

[24] Marchiori A Mosena L Prandoni P . Superficial vein thrombosis: risk ractors, diagnosis, and treatment. Semin Thromb Hemost. 2006;32(7):737 743. 10.1055/s-2006-951459) 17024602

[25] Litzendorf ME Satiani B . Superficial venous thrombosis: disease progression and evolving treatment approaches. Vasc Health Risk Manag. 2011;7:569 575. 10.2147/VHRM.S15562) 21966221 PMC3180510

[26] Battistelli S Stefanoni M Lorenzi B et al. Coagulation factor levels in nonmetastatic colorectal cancer patients. Int J Biol Markers. 2008;23:36 41. 18409149

[27] Minnema MC Fijnheer R De Groot PG Lokhorst HM . Extremely high levels of von Willebrand factor antigen and of procoagulant factor VIII found in multiple myeloma patients are associated with activity status but not with thalidomide treatment. J Thromb Haemost. 2003;1(3):445 449. 10.1046/j.1538-7836.2003.00083.x) 12871448

[28] Gulledge AA Rezaee F Verheijen JH Lord ST . A novel transgenic mouse model of hyperfibrinogenemia. Thromb Haemost. 2001;86(2):511 516. 10.1055/s-0037-1616079) 11521996

[29] Tracy RP Bovill EG Fried LP et al. The distribution of coagulation factors VII and VIII and fibrinogen in adults over 65 years. Results from the cardiovascular Health Study. Ann Epidemiol. 1992;2(4):509 519. 10.1016/1047-2797(92)90100-5) 1342301

[30] Stone MC Thorp JM . Plasma fibrinogen—a major coronary risk factor. J R Coll Gen Pract. 1985;35(281):565 569. 4093900 PMC1961456

[31] Wilhelmsen L Svärdsudd K Korsan-Bengtsen K Larsson B Welin L Tibblin G . Fibrinogen as a risk factor for stroke and myocardial infarction. N Engl J Med. 1984;311(8):501 505. 10.1056/NEJM198408233110804) 6749207

[32] Kannel WB Wolf PA Castelli WP D’Agostino RB . Fibrinogen and risk of cardiovascular disease: the Framingham study. JAMA. 1987;258(9):1183 1186. 3626001

[33] Tracy RP Bovill EG Yanez D et al. Fibrinogen and factor VIII, but not factor VII, are associated with measures of subclinical cardiovascular disease in the elderly: results from the cardiovascular Heath Study. Arterioscler Thromb Vasc Biol. 1995;15(9):1269 1279. 10.1161/01.atv.15.9.1269) 7670938

[34] Meade T The epidemiology of haemostatic and other variables in coronary artery disease. In: Verstraete M Vermylen J Lijnen H Arnout J eds. Thromb Haemost. Leuven: International Society on Thrombosis and Haemostasis and Leuven University Press; 1987:37 59.

[35] Kannel WB D’Agostino RB Belanger AJ . Update on fibrinogen as a cardiovascular risk factor. Ann Epidemiol. 1992;2(4):457 466. 10.1016/1047-2797(92)90095-8) 1342296

[36] Tracy RP Bovill EG . Thrombosis and cardiovascular risk in the elderly. Arch Pathol Lab Med. 1992;16:1307 1312. 1456876

[37] Tsai AW Cushman M Rosamond WD et al. Coagulation factors, inflammation markers, and venous thromboembolism: the longitudinal investigation of thromboembolism etiology (LITE). Am J Med. 2002;113(8):636 642. 10.1016/s0002-9343(02)01345-1) 12505113

[38] De Stefano V Rossi E Paciaroni K Leone G . Screening for inherited thrombophilia: indications and therapeutic implications. Haematologica. 2002;87(10):1095 1108. 12368166

